# *CACNA1C* rs1006737 SNP increases the risk of essential hypertension in both Chinese Han and ethnic Russian people of Northeast Asia

**DOI:** 10.1097/MD.0000000000024825

**Published:** 2021-02-26

**Authors:** Hao Zhang, Boris Pushkarev, Jiexin Zhou, Yuyuan Mu, Olga Bolshakova, Sandeep Shrestha, Ningning Wang, Baiyu Jian, Ming Jin, Keyong Zhang, Mingyu Cong, Jicheng Liu, Yuri Vitkovsky, Changchun Qiu

**Affiliations:** a Institute of Polygenic Disease, Qiqihar Medical University, No. 333 Bukui Street, Jianhua District, Qiqihar, Heilongjiang Province , PR China; b Chita State Medical Academy, 39a Gorky Street, Chita, Russian Federation; c Institute of Basic Medical Sciences, Chinese Academy of Medical Sciences Peking Union Medical College (CAMS/PUMC), Beijing, PR China.

**Keywords:** biracial study, *CACNA1C* gene, Chinese Han and ethnic Russian people, essential hypertension, genetics, specific latitudes

## Abstract

Voltage-gated Ca^2+^ channels play a key role in the regulation of arterial tone and blood pressure. The aim of this study was to determine whether the association of calcium voltage-gated channel subunit alpha1 C *(CACNA1C)* rs1006737 with essential hypertension (EH) exists in both Chinese Han and ethnic Russian populations of Northeast Asia. We used a case-control study of 2 ethnic groups in the same latitude geographical area to investigate the association between the susceptibility of EH and rs1006737 polymorphism. A total of 1512 EH patients and 1690 controls in Chinese Han people (Heilongjiang Provence, China), 250 EH patients, and 250 controls in ethnic Russian people (Chita, Russia), participated in this study. All participants were genotyped using the TaqMan SNP genotyping assay (Agena Company). Baseline characteristics and the minor allele frequencies of rs1006737 vary substantially among common Chinese Han and ethnic Russian people. Allele A was found to be a risk factor for EH in Chinese Han [(odds ratio) OR 1.705, (confidence interval) 95% CI: 1.332–2.182, *P* < .001] and ethnic Russian (OR 1.437; 95% CI: 1.110–1.860, *P* = .006). The GA genotype was significantly associated with an increased risk of hypertension (OR 1.538, 95% CI: 1.188–1.991, *P* = .001) for Chinese Han people, and the AA genotype (OR 2.412, 95% CI: 1.348–4.318, *P* = .003) for ethnic Russian people. The results of this study indicate that the A allele of the variant rs1006737 in the *CACNA1C* gene may be a useful genetic marker for EH risk prediction in Chinese Han and ethnic Russian populations.

## 1 Introduction

Essential hypertension (EH) is the consequence of an interaction between genetic, environmental, and lifestyle factors and does not follow Mendelian genetic law.^[1]^ According to Basu et al, there was significant prevalence of hypertension in adults varied from 39% in the Chinese population to 52% in the Russian population.^[2]^ Hypertension is a major contributor to health burden in both China and Russia due to its large impact on a number of cardiovascular diseases.^[3–5]^ A genetic association study is difficult to repeat in 2 races due to enrollment and environmental factors; however, statements about geographical factors such as latitude, solar radiation, and ambient temperature are often not referenced.^[6,7]^ It is plausible that geographic factors impact the results of a research study. Therefore, to determine shared genetic susceptibility of genes, a case-control study of Chinese and Russian people in the same geographical area may be a better design to minimize the impact of geographical factors.

Calcium channel is an important target of anti-hypertensive drugs, and voltage-gated Ca^2+^ channels serve as key transducer coupling changes in cell surface membrane potential with local intracellular calcium (Ca^2+^) pathways.^[8]^ The L-type α1c subunit plays a central role in the regulation of cardiac function and blood pressure. The human alpha 1C subunit of the L-type voltage-gated calcium channel (*CACNA1C)* gene is a potential candidate gene for EH development,^[9]^ which is located at chromosomal position 12p13.33 (chr12: 1,896,712–2,770,789; NCBI Genome Data Viewer; GRCh38.p12). EH has been shown to be heritable and shared genetic risk components with other diseases. Previous studies have shown that patients with psychiatric disorders, such as bipolar disorder, schizophrenia, or major depression, have a greater risk for hypertension than those without such pathologies.^[10]^*CACNA1C* gene rs1006737 polymorphism was reported to be associated with psychiatric disorders.^[11–13]^ It is not yet known whether EH and psychiatric disorders more broadly share biological underpinnings. Therefore, we hypothesized that the allele of rs1006737 might contribute to elevated blood pressure (BP) over time in humans.

From 2016, we carried out Sino-Russian scientific research on cardiovascular and cerebrovascular diseases in the Northeast Asia region. The results of our previous study showed an association of *CACNA1C* rs1006737 polymorphism with EH in ethnic Russians. The aim of this study was to assess the association between human *CACNA1C* gene rs1006737 and susceptibility of EH in different races, including Chinese Han and ethnic Russian people in the same geographical area, further supporting the hypothesis that EH and psychiatric disorders share the same SNP locus in genetic susceptibility.

## 2 Methods

### 2.1 Subjects

**Figure 1:**
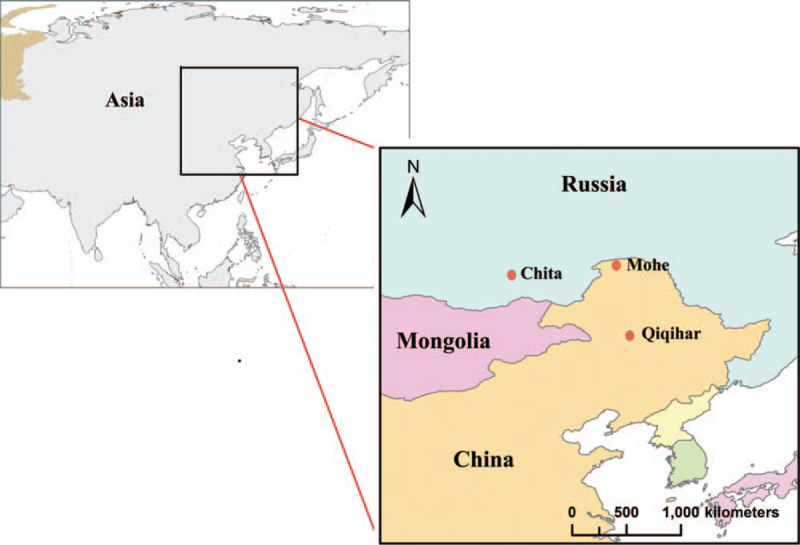
Northeast Asia region.

In this study, all Chinese Han patients with EH were recruited from the No.2 affiliated hospital of Qiqihar Medical University and Mohe People's Hospital (Heilongjiang province, China), and healthy volunteers were enrolled by health examination in the Heilongjiang region from December 2016 to July 2019. Russian participants were enrolled from City Clinical Hospital No. 1 and Railway Clinical Hospital at Chita-2 Station JSC RZhD (Chita, Zabaikalsky Kray, Russia) from September 2010 to June 2017. All Russian participants were of Caucasian origin, from the Chita population. Informed consent was obtained from each participant, and all protocols were approved by the Medical Ethics Committee of Qiqihar University and Chita State Medical Academy. All Chinese participants were of northern Han Chinese within 3 generations, and are currently residing in Qiqihar (47° 21 0” N, 123° 55” 0” E) and Mohe (52° 58 19” N, 122° 32 20” E) region of Heilongjiang province. All Russian participants were from Chita within 3 generations, and are currently residing in Chita (52° 03 0” N, 113° 28” 0” E) of Zabaykalsky Krai, in the southeast of Russia (Fig. 1).

The measuring data of blood pressure came from Riva-Rocci sphygmomanometer. After resting for ten minutes, the blood pressure of the subjects was measured 5 minutes apart there times on the right arm by experienced doctors according to the standard protocol recommended by the American Heart Association.^[14]^ Three consecutive values of blood pressure were averaged for matching inclusion criteria and analysis. Body mass index (BMI) was calculated by height and weight, which were obtained without shoes and subjects wearing light clothes.

In detail, a total of 1512 unrelated Chinese Han patients (744 males and 768 females) with EH and 1690 Chinese Han normotensive subjects (790 men and 900 women) were included in this investigation after completion of matching inclusion criteria. Russian participants comprised 250 patients (126 males and 124 females) and 250 healthy individuals (123 males and 127 females).

Subjects aged >18 years, who consistently had systolic blood pressure (SBP) >140 mm Hg and/or diastolic blood pressure (DBP) >90 mm Hg, or with a history of hypertension were diagnosed as hypertensive. The control group was composed of subjects aged >40 years old with blood pressure SBP <120 mm Hg and DBP <80 mm Hg, without personal or family history of hypertension and other cardiovascular diseases, and patients with minor illness without hypertension, hyperlipidaemia, diabetes mellitus, tumor in their previous records. Secondary hypertension and pregnancy were excluded from this study.

### 2.2 SNP selection and genotyping

EH and psychiatric disorders such as schizophrenia and bipolar disorder may share the same susceptibility locus,^[15]^ and it is not yet known whether there is biological overlap in etiology.

Candidate gene studies have implicated aberrations in ion channels and ion channel regulation in EH. Genome-wide association studies (GWAS) have detected more than 50 BP loci, most previously unsuspected in EH.^[16]^ The calcium channel is an important target in the treatment of hypertension and calcium channel blockers are widely used as antihypertensive agents. The *CACNA1C* gene, located on chromosome 12p13.33, encodes an alpha-1 subunit of a voltage-dependent calcium channel that mediates the influx of calcium ions into the cell upon membrane polarization. GWAS have identified rs1006737 in the α1c-subunit of the L-type calcium channel (*CACNA1C)* gene as a susceptibility locus for psychiatric disorders.^[17,18]^

Many gene polymorphism loci have been extensively studied in various populations, but the results of these studies are often inconsistent in different races, so we chose rs1006737 as a biomarker to conduct a case-control study between Chinese Han and ethnic Russian people in the same geographical region.

Blood samples were collected from all participants and anticoagulated with 2% ethylenediaminetetraacetic acid. Genomic DNA was extracted from peripheral blood leukocytes using a DNA extraction kit (ComWin Biotech Company, Beijing) and stored at –80°C. Genotyping was performed using the TaqMan SNP genotyping assay (Agena Company). Primers and probes were chosen from the information available on the ABI website.

### 2.3 Statistical analysis

Data management and statistical analysis were performed with Statistical Product and Service Solutions (SPSS version 21.0). The quantitative variables are presented as the mean (SD), which are normally distributed. Hardy-Weinberg equilibrium (HWE) was tested in the healthy group using a Chi-Squared test. The difference in continuous data was compared using the Mann–Whitney *U* test. The Chi-Squared test was used to calculate categorical variables. After single-factor analysis, multiple logistic regression models were used to analyze the independent effect of each genetic variant on the risk of EH. The allele frequency, genotype, and factor between the groups were examined by unconditional logistic regression analysis. A *P* value of less than .05 was considered statistically significant.

## 3 Results

### 3.1 Baseline characteristics

A total of 3202 Chinese Han (1512 patients with EH and 1690 controls) and 500 ethnic Russian (250 patients with EH and 250 controls) individuals participated in this study. Table 1 shows the baseline characteristics between the control and patients with EH between the Chinese Han and ethnic Russian population. There were significant differences in BMI, SBP, DBP, and pulse pressure between the 2 groups in the Chinese Han population (*P* < .05). In the Russian part of Table 1, compared with Chinese Han people, Russian people have similar results in BMI, SBP, and DBP (*P* < .01). However, no statistical significance was found in sex or weight (*P* > .05) in the ethnic Russian population. There were significant differences in Cholesterol (CHOL), Triglyceride (TG), and Glucose (GLU) between the 2 groups in Chinese Han people (*P* < .05). However, no statistical significance was found in CHOL and GLU (*P* > .05) in the ethnic Russian population. Baseline characteristics of healthy Chinese Han and ethnic Russian individuals aged 40–50 years are shown in Table 2.

**Table 1:** Baseline characteristics between control and patients with Essential Hypertension.

	Chinese Han			Ethnic Russian		
Characteristic	Hypertension (n = 1512)	Control (n = 1690)	*P*	Hypertension (n = 250)	Control (n = 250)	*P*
Age, years old	52.62 (8.22)	52.75 (7.04)	.072	43.65 (5.37)	41.92 (4.18)	.005
Sex (male/female)	744/768	790/900	.325	126/124	123/127	.788
Height, cm	162.42 (8.84)	163.42 (9.32)	.001	170.67 (8.74)	174.52 (6.87)	.035
Weight,kg	68.19 (13.46)	63.17 (10.07)	<.001	86.52 (17.42)	80.97 (12.65)	.231
BMI, kg/m^2^	25.72 (3.78)	23.63 (3.14)	<.001	29.74 (5.92)	26.52 (3.54)	.006
SBP, mm Hg	146.50 (16.47)	109.40 (8.30)	<.001	150.27 (7.74)	115.45 (4.20)	<.001
DBP, mmHg	92.70 (9.51)	73.17 (5.73)	<.001	94.21 (5.54)	71.74 (3.12)	<.001
Pulse pressure, mm Hg	53.79 (14.60)	36.24 (6.60)	<.001	56.07 (8.66)	49.71 (4.83)	<.001
CHOL, mmol/L	5.71 (1.17)	5.23 (1.03)	<.001	5.14 (0.98)	5.19 (0.92)	.762
TG, mmol/L	1.84 (1.37)	1.46 (1.01)	<.001	2.19 (1.25)	1.95 (0.92)	.037
GLU, mmol/L	5.02 (1.45)	4.71 (0.79)	<.001	5.04 (0.41)	5.08 (0.52)	.159
CRE, μmol/L	63.84 (8.45)	64.82 (9.02)	.067	94.54 (11.63)	90.79 (13.32)	<.001

Values are given as mean (SD). n = number of subjects, *P* = *P* value.

**Table 2:** Baseline characteristics of healthy Chinese Han and ethnic Russian aged 40–50 years old.

	Whole			Male			Female		
Characteristics	Chinese Han (n = 780)	Russian (n = 181)	*P*	Chinese Han (n = 350)	Russian (n = 84)	*P*	Chinese Han (n = 430)	Russian (n = 97)	*P*
Age, years old	46.60 (2.61)	43.88 (2.92)	<.001	45.91 (3.18)	43.94 (2.90)	<.001	47.16 (1.86)	43.84 (2.95)	<.001
Sex (male/female)	350/430	84/97	.708	–	–	–		–	–
Height, cm	165.14 (8.95)	173.45 (6.32)	<.001	172.67 (6.48)	174.73 (5.16)	.135	159.01 (5.24)	162.33 (4.51)	.149
Weight, kg	65.53 (9.98)	79.28 (12.14)	<.001	69.53 (8.83)	81.04 (11.25)	<.001	62.28 (9.69)	64.00 (9.54)	.318
BMI, kg/m^2^	24.01 (3.03)	26.30 (3.51)	<.001	23.29 (2.43)	26.51 (3.38)	<.001	24.59 (3.33)	24.43 (4.88)	.965
SBP, mm Hg	107.84 (8.58)	121.24 (4.14)	<.001	108.15 (8.68)	124.36 (2.61)	<.001	107.58 (8.50)	118.54 (3.22)	<.001
DBP, mmHg	73.15 (5.71)	71.73 (3.16)	<.001	73.52 (5.70)	72.70 (3.17)	.017	72.85 (5.71)	70.89 (2.91)	<.001
Pulse Pressure, mm Hg	34.68 (6.37)	49.51 (4.61)	<.001	34.63 (7.09)	51.66 (4.16)	<.001	34.73 (5.73)	47.65 (4.16)	<.001
CHOL, mmol/L	5.26 (1.01)	5.22 (0.94)	.407	5.23 (0.95)	4.72 (0.60)	<.001	5.29 (1.05)	5.66 (0.96)	.001
TG, mmol/L	1.48 (0.87)	1.92 (0.91)	<.001	1.46 (0.93)	1.88 (0.76)	<.001	1.50 (0.82)	1.95 (1.02)	<.001
GLU, mmol/L	4.78 (0.76)	5.08 (0.49)	<.001	4.62 (0.54)	5.29 (0.43)	<.001	4.90 (0.88)	4.89 (0.47)	.034
CRE, μmol/L	64.38 (8.04)	90.62 (13.18)	<.001	64.22 (8.56)	94.62 (9.44)	<.001	64.51 (7.62)	87.12 (14.94)	<.001

Values are given as mean (SD). MAF = minor allele frequency, n = number of subjects, *P* = *P* value.

A multiple logistic regression model showed that CHOL [odds ratio OR 3.071; confidence interval 95% CI 2.577–3.659], BMI (OR 2.467; 95% CI 2.136–2.850), rs1006737 (OR 1.600; 95% CI 1.228–2.084), TG (OR 1.543; 95% CI 1.313–1.814), and GLU (OR 1.571; 95% CI 1.327–1.860) to be significantly correlated with EH in the Chinese Han population. TG (OR 1.612; 95% CI 1.123–2.313) and rs1006737 (OR 1.386; 95%CI 1.059–1.813) were independent risk factors in the ethnic Russian population.

Unconditional logistic regression model showed correlative risk factors for EH (Table 3). Compared with females, males do not have an increased risk for EH, but higher values of age, BMI, CHOL, TG, and GLU could increase the risk for EH.

**Table 3:** Correlative Risk Factors for Essential hypertension.

	Chinese Han		Ethnic Russian	
Factor	OR (95% CI)	*P*	OR (95% CI)	*P*
Male	1 (reference)		1 (reference)	
Female	0.906 (0.789–1.041)	.164	0.953 (0.671–1.353)	.788
Age <50	1 (reference)		1 (reference)	
Age ≥50	1.145 (0.990–1.325)	.068	6.180 (2.346–16.283)	<.001
BMI <25	1 (reference)		1 (reference)	
BMI ≥25	2.563 (2.217–2.964)	<.001	2.409 (1.063–5.460)	.035
CHOL <6.22	1 (reference)		1 (reference)	
CHOL ≥6.22	4.415 (3.806–5.121)	<.001	1.027 (0.654–1.611)	.908
TG <2.26	1 (reference)		1 (reference)	
TG ≥2.26	2.417 (2.062–2.883)	<.001	1.635 (1.130–2.366)	.009
GLU <6.1	1 (reference)		1 (reference)	
GLU ≥6.1	5.116 (3.723–7.029)	<.001	4.205 (1.386–12.762)	.011

The odds ratios (ORs), 95% confidence intervals (CIs), and *P* values were obtained by unconditional logistic regression analysis.

### 3.2 Single-locus analysis

The distribution of the *CACNA1C* genotype conformed to Hardy-Weinberg equilibrium (HWE) with *P* = .299 (Chinese Han control people) and *P* = .304 (Russian control people), respectively.

**Figure 2:**
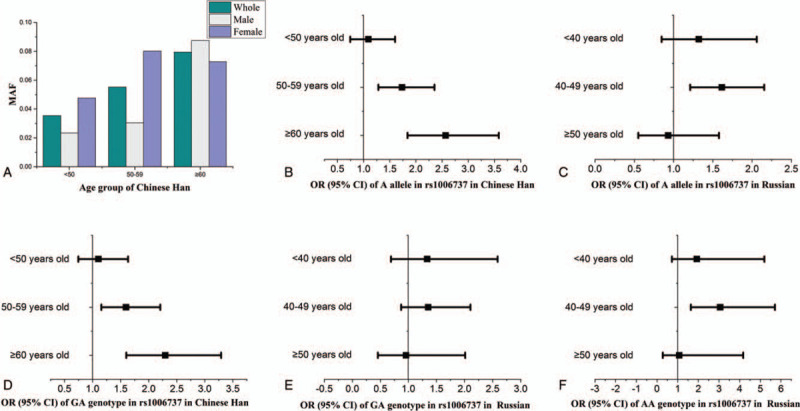
A: The relationship among age, sex and MAF of 1006737 in Chinese hypertension group. B–C: The relationship between age and OR values of the allele in rs1006737. D–F: The relationship between age and OR values of genotype in rs1006737.

The MAF of rs1006737 increased with advancing age in Chinese Han hypertension and control groups, from 0.0294 (age < 40 years) to 0.0795 (age >60 years) in the hypertension group, from 0.0242 (age <50 years old) to 0.0571 (age >60 years old) in the control group, the results were the same in the male and female groups (Fig. 2 A). There were similar trends in ethnic Russians.

Single-locus analyses of genotype and allele distributions in normotensive and EH patients for these SNPs are summarized in Table 4. Allele A of rs1006737 was found to be a risk factor for EH (odds ratio [OR] 1.705, 95% confidence interval [95%CI] 1.332–2.182, *P* < .001) (Chinese Han) and (OR = 1.437; 95% CI: 1.110–1.860, *P* = .006) (ethnic Russian).

**Table 4:** Genotypes of rs1006737in *CACNA1C* gene and the risk of essential hypertension in Chinese Han people.

Race	Genotype	Hypertension	Control	OR (95%CI)	*P* (χ2)	OR^∗^ (95%CI)	*P*^∗^
Chinese Han	MAF	0.0542	0.0325				
	Allele frequency G:A	0.946:0.054	0.967:0.033	1.705 (1.332–2.182)	<.001 (18.33)		
	GG	1356 (89.7)	1580 (93.5)	1.000 (reference)		1.000 (reference)	
	GA	148 (9.8)	110 (6.5)	1.568 (1.212–2.027)	.001	1.538 (1.188–1.991)	.001
	AA	8 (0.5)	0 (0.0)	–	–	–	–
	Dominant (GG vs GA+AA)			1.652 (1.281–2.132)	<.001	1.618 (1.253–2.089)	<.001
	Recessive (GG+GA vs AA)			–	–	–	–
Ethnic Russian	MAF	0.410	0.326				
	Allele frequency G:A	0.590:0.410	0.674:0.326	1.437 (1.110–1.860)	.006 (7.58)		
	GG	89 (35.6)	110 (44.0)	1.000 (reference)		1.000 (reference)	
	GA	117 (46.8)	117 (46.8)	1.236 (0.846–1.806)	.273	1.308 (0.887–1.927)	.175
	AA	44 (17.6)	23 (9.2)	2.364 (1.328–4.208)	.003	2.412 (1.348–4.318)	.003
	Dominant (GG vs GA+AA)			1.421 (0.992–2.037)	.055	1.496 (1.036–2.162)	.032
	Recessive (GG+GA vs AA)			2.108 (1.230–3.612)	.007	2.084 (1.213–3.582)	.008

∗ adjusted by age, sex and BMI. MAF = minor allele frequency.

The GA genotype frequency of rs1006737 was significantly associated with an increased risk of hypertension (OR = 1.538, 95% CI: 1.188–1.991, *P* = .001) for Chinese Han people. The AA genotype frequency of rs1006737 was significantly associated with an increased risk of hypertension (OR = 2.412, 95% CI: 1.348–4.318, *P* = .003) for ethnic Russian people. In the dominant model, GA+AA genotype carriers had a 61.8% increased risk of EH relative to those with the GG genotype (95% CI: 1.253–2.089, *P* < .001) for Chinese Han people and 49.6% increased risk (95% CI: 1.036–2.162, *P* = .032) for ethnic Russian people. In the recessive model, AA genotype carriers had a 108.4% increased risk of EH relative to those with GG+GA genotype (95% CI: 1.213–3.582, *P* = .008) for ethnic Russian people. Following advanced age, the GA genotype had higher OR values in the Chinese Han hypertension group, OR = 1.105 (age <50 years), OR = 1.597 (aged 50–59 years), OR = 2.295 (age >60 years old). Similar trends were observed for AA genotype in ethnic Russian people: OR = 1.932 (age <40 years old), OR = 3.060 (aged 40–49 years old), OR = 1.072 (age >50 years old) (Fig. 2 B-F)

## 4 Discussion

EH is a complex and polygene genetic disease, evidence suggests that polymorphism of gene has been associated with EH in various populations.^[19–23]^ Our study provides a China-Russia biracial view of hypertension in the same geographical region for the first time. Our main findings were that

1. allele A of rs1006737 was found to be a risk factor for EH in both races, EH and psychiatric disorder might share this locus in genetic susceptibility;
2. baseline characteristics vary substantially, and MAFs in rs1006737 of 2 races range from 0.0192 to 0.3287 among healthy Chinese Han and ethnic Russian populations aged 40 to 50 years, ethnic Russian have higher MAF;
3. MAF of rs1006737 was positively associated with age in Chinese Han people.

Asian and white adults were reported to have higher rates of diagnosed hypertension from an analysis of the 2010 to 2016 National Health Interview Survey.^[24]^ Li et al ^[25]^ showed that 27.8% of Chinese adults were hypertensive in 2013 to 14, as assessed by the Burden of Hypertension in China: A nationally representative survey of 174,621 adults. The average prevalence of hypertension in Russia was estimated at 44% based on the data of 10 regions from the ESSE-RF study.^[4]^ Blood pressure level and the prevalence of hypertension vary widely throughout the world, and geographical latitude, solar radiation, and ambient temperature factors have been cited as possible explanations for this variance. Geographical latitude was positively associated with the prevalence of hypertension in Cabrera's study.^[26]^ There is a relatively high incidence of hypertension in the Heilongjiang region (China), which has the highest latitude in China.

From baseline characteristics of healthy populations aged 40 to 50 years, we have proven that there are large differences in demographic data between the 2 races, especially in male populations. From the results of this study, ethnic Russians suffer from EH earlier than Chinese Han in the Northeast Asia region, which involves a mix of genetic and environmental factors.

Although the MAFs of Chinese Han and ethnic Russian populations are different, the trend of the A allele of rs1006737 risk is similar in this study. These results come from the A allele of rs1006737, which is a risk factor for EH, even if MAF rs1006737 is lower in Chinese Han people. Although genome-wide studies have provided valuable insights into the genetic basis of EH, candidate genes for EH are more powerful for detecting risk variants than to detect protective variants, especially when MAF <0.05.^[27]^

EH and psychiatric disorders shared the rs1006737 locus in genetic susceptibility from the results of our study. Some patients with psychiatric disorders also take antihypertensive drugs to treat their mental illness.^[28]^ Calcium-channel blockers are widely used to treat hypertension because they are more effective than other types of antihypertensive agents.^[29]^*CACNA1C* gene encodes protein is expressed in neurons and other electrically excitable tissues (heart, smooth muscle), sensory, and endocrine cells,^[30]^ which play an important role in regulating BP.

Shared genetic susceptibility suggests that some complex diseases might share the same pathological process in the early stages of the disease. In a genome-wide association study, the present study demonstrated that *the human CACNA1C* gene rs1006737 is associated with patients with psychiatric disorders.^[11]^ While researchers have achieved satisfactory experimental results, people may have overlooked biological hints that complex diseases might share the same susceptibility locus and pathological process. There is little evidence that hypertension and psychiatric disorders share the same genetic basis for common diseases. For most common diseases, polygenic inheritance plays a greater role than rare monogenic mutations.

There is the same susceptibility locus of hypertension rs1006737 in *the human CACNA1C* gene, although the MAFs of the 2 races are different. An allele of rs1006737 in Chinese Han populations shows more power to detect the risk of EH than ethnic Russian people. The OR values of the A allele become higher following the increase in age. There is a similar trend in ethnic Russian people. Because of the limited sample size in Russian people, the OR value of the allele shows a different trend in the age >50 group.

This study had several strengths: to minimize the impact of geographical factors, the use of 2 cohorts from China and Russia in similar latitudes in the Northeast Asia region, and the population-based design of data collection for 2 populations. However, our study also had some limitations. Our study sample was relatively small, which are hardly representative of the whole Chinese Han and Russian population, but effects of biogeographic and climatic factors were considered. We selected only 1 SNP locus as the research object, which is insufficient to explain the pathogenesis of a polygenic trait such as hypertension. However, other purpose of designing experiments like this is to remind other researchers that people often overlook the influence of geographic factors on disease. This, on the other hand, may explain why the results of genetic association studies are difficult to repeat. Environmental factors such as geography and lifestyle may influence complex diseases more than genetic factors. Russian cohorts had a small sample size and relatively concentrated age, and the control group was younger. We consider that this might be due to a small population in Russia. Participants using antihypertensive drugs were classified as hypertensive. Ambulatory BP was not performed, which might have caused errors in the estimation of white coat and masked hypertensive subjects. These factors might affect the average blood pressure levels.

### 4.1 Perspectives

This kind of design provides a new view of genetic association studies; rs1006737 was also shown to contribute to EH and psychiatric disorders, further supporting the hypothesis that EH and psychiatric disorders show biological overlap in genetic susceptibility. EH and psychiatric disorders are now recognized as leading causes of morbidity and affect the human population around the world. Increasing evidence suggests an overlap in genetic susceptibility across the traditional classification systems of disease. Future identification of EH and psychosis susceptibility genes will have a major impact on our understanding of disease pathophysiology and will lead to changes in classification and the clinical practice of disease.^[31]^

Achieving precision therapy requires molecular level detection, and new molecular level experimental results may promote disease reclassification. We will treat seemingly irrelevant diseases together. Although a key public health need is to identify individuals at high risk for common disease to enable enhanced screening or preventive therapies, it has not yet been possible to use polygenic predictors to identify individuals at high risk compared to monogentic disease. Multiple SNP tests may be used to improve risk prediction models in conjunction with clinical assessments. In the future, humans need to create a type of polygenic score to identify individuals at clinically significantly increased risk or multivariable risk prediction model to predict the probability of developing EH at the gene level. Shared susceptibility may be a key hint to find a valued locus of SNP, and previous studies have shown that common susceptibility variants are shared in common complex disorders.

## Acknowledgments

We thank the subjects for participating in our study. We thank Emelyanov Arthur, Romanyuk Svetlana. We gratefully acknowledge the assistance of the clinical, field, and laboratory staff that made this work possible.

## Author contributions

**Conceptualization:** Yuri Vitkovsky, Changchun Qiu.

**Data curation:** Mingyu Cong.

**Formal analysis:** Ningning Wang.

**Funding acquisition:** Hao Zhang, Changchun Qiu.

**Investigation:** Jiexin Zhou, Yuyuan Mu, Olga Bolshakova.

**Methodology:** Baiyu Jian.

**Resources:** Keyong Zhang, Jicheng Liu.

**Software:** Ming Jin.

**Writing – original draft:** Hao Zhang, Boris Pushkarev.

**Writing – review & editing:** Hao Zhang, Sandeep Shrestha, Changchun Qiu.
